# Copy‐Paste Augmentation Improves Automatic Species Identification in Camera Trap Images

**DOI:** 10.1002/ece3.72357

**Published:** 2025-11-05

**Authors:** Cédric S. Mesnage, Andrew Corbett, Jake Curry, Sareh Rowlands, Anjan Dutta, Richard Everson, Benno I. Simmons

**Affiliations:** ^1^ Institute for Data Science and Artificial Intelligence University of Exeter Exeter UK; ^2^ Department of Computer Science University of Exeter Exeter UK; ^3^ Centre for Ecology and Conservation University of Exeter Penryn UK; ^4^ Institute for People‐Centred Artificial Intelligence University of Surrey Guildford UK

**Keywords:** AI, augmentation, camera trap, computer vision, machine learning, monitoring, serengeti, vertebrates

## Abstract

Effective conservation requires effective biodiversity monitoring. The pace of global biodiversity change far outstrips the ability of manual fieldwork to monitor it. Therefore, technological solutions, like camera traps, have emerged as a crucial way to meet biodiversity monitoring needs. Camera traps produce vast amounts of data, and so AI is increasingly used to label images with species identities. However, AI struggles to identify species from new locations that are not part of the training data (‘generalisation’). Resolving this is crucial for the promise of automated biodiversity monitoring to be realised. Here we use ‘copy‐paste’ augmentation to help resolve the generalisation challenge. Copy‐paste augmentation refers to isolating animal ‘segments’ from existing images and pasting the segments onto novel backgrounds to create new, synthetic images that are then used as part of the training data. Theoretically, this could make a model agnostic to backgrounds and therefore more able to generalise to unseen locations. While generation of synthetic images is commonly used as an augmentation method in other fields, such as medicine, it has not been used before in biodiversity science. We found that copy‐paste augmentation improved the ability of AI to identify species in new, unseen locations by 8% ± 2%. There was species‐level variation in improvement, but the vast majority of species benefited from the approach. We found mixed results when using copy‐paste augmentation on models trained with very small numbers of images (1–8 per species). Copy‐paste augmentation improves the ability of AI models to generalise to new, unseen locations. Our method also shows promise for resolving the challenge of long‐tailed camera trap data. AIs perform poorly on species in the ‘long tail’ of these distributions because there are very few images to train on. Copy‐paste augmentation can help rebalance datasets by adding synthetic images of underrepresented species. Overall, our results suggest a promising role for augmentation methods that generate new, synthetic images in biodiversity science. Ecologists and conservationists must move beyond simple augmentation methods, such as image transformations, if we are to resolve key challenges in species identification AI.

## Graphical abstract

Effective biodiversity monitoring requires AI to process the vast data from camera traps, but AI struggles to generalise to new locations. This study explores ‘copy‐paste’ augmentation—creating synthetic images by pasting animal segments onto new backgrounds—to improve AI's species identification. Results show an 8% improvement in AI generalisation, particularly for underrepresented species, highlighting the potential of advanced augmentation methods in biodiversity science.

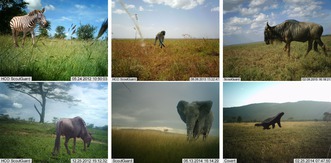

## 1 Introduction

Monitoring biodiversity is essential to track progress towards policy objectives and to assess the effectiveness of conservation actions. Traditionally, biodiversity monitoring relies on manual fieldwork, where researchers sample some aspect of biodiversity across space and/or time. However, this kind of fieldwork is both expensive and time‐consuming and thus scales poorly (Caughlan and Oakley 2001). While traditional fieldwork will always play a role in ecology, it alone cannot meet the growing need for up‐to‐date information about the state of global biodiversity (Leadley et al. 2022).

Technology offers a possible solution to this problem, through the advent of passive monitoring techniques, such as camera traps. Camera traps—motion‐ or heat‐activated cameras that capture images of wild animals—have great potential to help monitor biodiversity at scale: their low cost allows them to be deployed in vast arrays, collecting data on wildlife locations and behaviour across long time spans and large spatial extents (Swanson et al. 2015). Camera traps are already widely used and demonstrably useful, producing essential insights into population sizes, species richness, animal behaviour, disease spread, migration patterns, movement ecology, predator–prey interactions and conservation management (Delisle et al. 2021).

One of the biggest barriers to harnessing the full potential of camera traps, however, is that processing the large amounts of data they collect remains a manual task: humans must view tens of thousands, or even millions, of images and identify any species that occur in each image. This work is extremely time‐consuming and it can take multiple person‐years to label all images in a single dataset (Norouzzadeh et al. 2018).

To solve this problem, deep learning algorithms have been proposed to automate the identification of animals in camera trap images. These AI approaches have produced impressive results. For example, using the 3.2 million‐image ‘Snapshot Serengeti’ dataset, deep neural networks automatically identified animals correctly in 96.6% of images, representing a saving of 8.4 years of human effort (Norouzzadeh et al. 2018).

While these figures are impressive and highlight the potential for artificial intelligence to transform conservation biology, they may also be misleading. This is because the majority of camera trap AI studies only evaluate performance on images from locations seen during training (Shahinfar et al. 2020; Schneider et al. 2020; Tabak et al. 2019). Conversely, when algorithms have been tested on their ability to generalise to new, previously unseen locations, they perform significantly worse (Beery et al. 2018; Schneider et al. 2020). The panacea for this field is for biodiversity monitoring to be fully automated, based on AI that can accurately identify all species in any camera trap image from anywhere in the world. Generalisation to new locations is clearly central to this mission, and thus it was recently identified as one of the main unsolved problems in the field (Schneider et al. 2020).

Deep learning algorithms may struggle to generalise to new locations because models overfit to particular backgrounds (Schneider et al. 2020). Thus, when new backgrounds are encountered, algorithms are more likely to fail. Some studies have tried to remedy this problem by cropping images, such that they contain fewer background pixels and animals occupy more of the frame (Norouzzadeh et al. 2021). This approach has shown promise, with algorithms trained on cropped images having greater accuracy than those trained on full images (Norouzzadeh et al. 2021; Beery et al. 2018). However, cropping is not a perfect solution because background pixels still remain in the image, preventing algorithms from being truly decoupled from the environmental contexts on which they were trained.

Recently, it was proposed that segmentation approaches could be used to completely remove the background from camera trap images, leaving just the animal ‘segments’ (Schneider et al. 2020). Training datasets could then be augmented with generated images, comprising animal segments ‘pasted’ onto novel backgrounds (Ghiasi et al. 2021a). Theoretically, this approach could allow models “to become agnostic to backgrounds, and thus able to generalise to any unseen location” (Schneider et al. 2020). However, despite the immense potential of this approach, it has never been attempted.

Here we make the first such attempt, using segmentation to create novel ‘copy‐paste’ images to augment a large dataset of real camera trap images. We assess the ability of copy‐paste augmentation to improve the ability of algorithms to generalise to new, unseen locations. We find that this approach improves accuracy and conclude this could have important implications for future work building towards a general AI for global biodiversity monitoring.

## 2 Materials and Methods

### 2.1 Data

We analysed the Snapshot Serengeti dataset (Swanson et al. 2015), available at https://lila.science/datasets/snapshot‐serengeti. Snapshot Serengeti has a number of advantages: (i) it is the largest camera trap image dataset available; (ii) it has a large number of bounding box annotations, which are a relatively uncommon annotation, but which were essential for our study; and (iii) it has been used by other studies in related work, facilitating comparisons between approaches (e.g., Norouzzadeh et al. (2018, 2021)). Of the 7 million images in the dataset, 74,616 have bounding box annotations around individual animals, giving their position and species identity. The dataset covers 225 different locations over six seasons. Unfortunately, the species identity (hereafter referred to as “classes”) annotations are given for a sequence of three images, and not for a particular bounding box, making it impossible to know which class corresponds to which bounding box without further manual inspection. We therefore focused on images where a single class was identified to remove this uncertainty. Another issue with the dataset is that images from some locations have been rescaled, while their bounding boxes have not; images from these locations were removed. The list of removed locations can be found in the Table S1. We also removed images where the bounding boxes were within 200 px of the edge of the frame that is., where animals are partly outside the frame of the image. These images were excluded because they would lead to parts of animals being segmented, rather than an entire non‐occluded animal, which is then problematic when copy‐pasting as it may lead to parts of animals appearing in the middle of a synthetic image.

### 2.2 Monte Carlo Cross Validation

To test for transferability (the ability of our trained AI to generalise to new, unseen locations), and to estimate the statistical significance of our results, we apply Monte Carlo cross validation. The following experiment is reproduced *k* times. We first randomly sample locations, selecting 80% of the locations for training and 20% of the locations for testing. We use 10 images per class from the test locations as a validation set; this is used at each epoch to evaluate the training in terms of accuracy. We evaluate on the test set for each *k* once the training is finished (note that results shown below are averaged over the *k* iterations of the Monte Carlo cross validation; the standard error is provided to capture variability between iterations).

**FIGURE 1:**
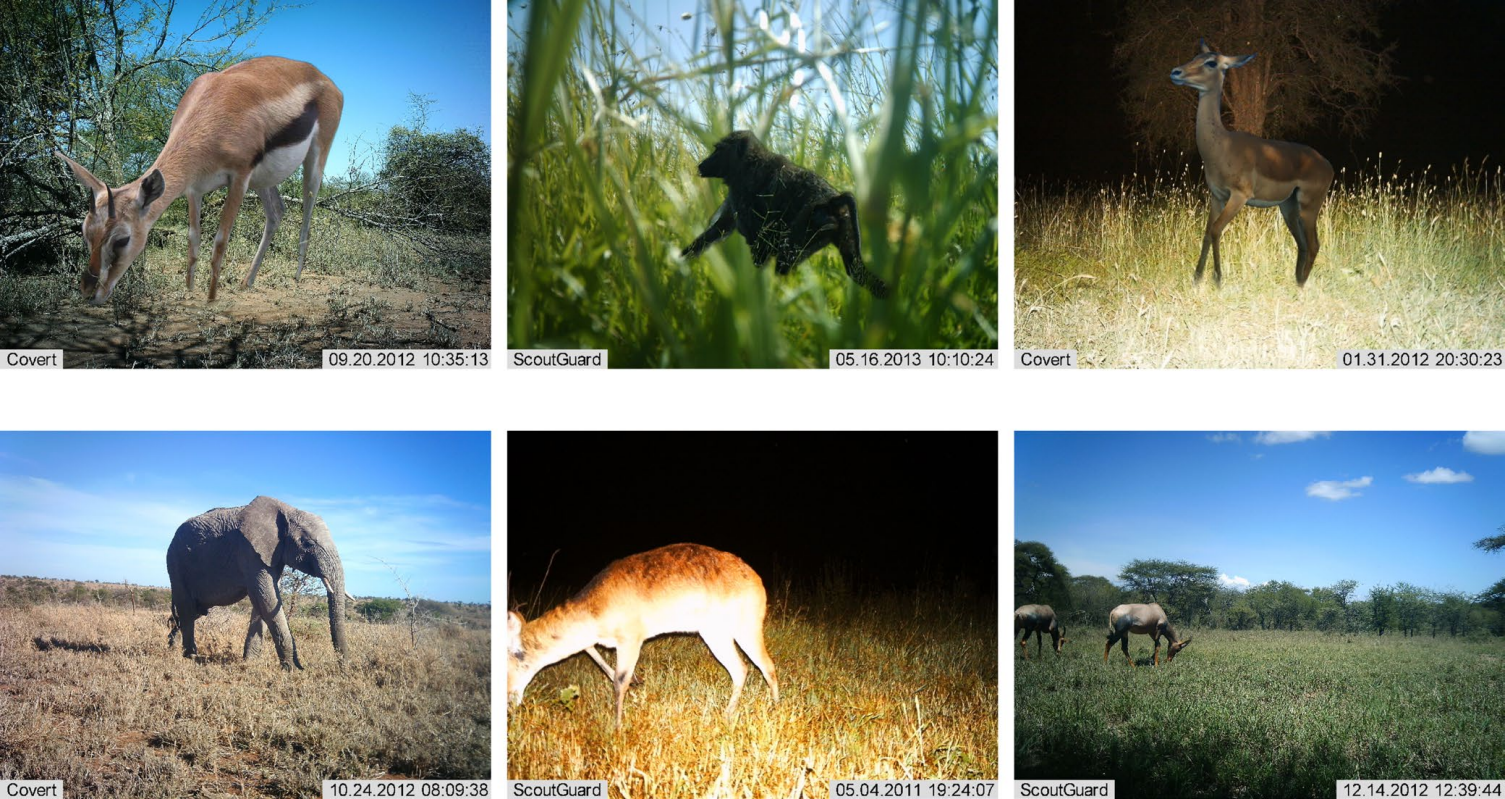
Examples of raw and generated training images. The top row shows generated training images resulting from our automated copy‐pasting strategy onto empty backgrounds. The bottom row shows raw, unedited images from the Snapshot Serengeti dataset.

We produce multiple training sets per iteration: a ‘raw’ training set with only real images, and ‘augmented’ training sets, which contain both real images and generated images. The augmented sets contain varying numbers of generated images, determined by the augmentation factor. An augmentation factor of 1 (indicated by aug_1 in plots) would describe an augmented set with an equal number of raw and generated images; an augmentation factor of 2 (indicated by aug_2 in plots) would describe an augmented set with twice as many generated images as raw images, and so on. Examples of raw and augmented images can be seen in Figure 1.

### 2.3 Image Segmentation

**FIGURE 2:**
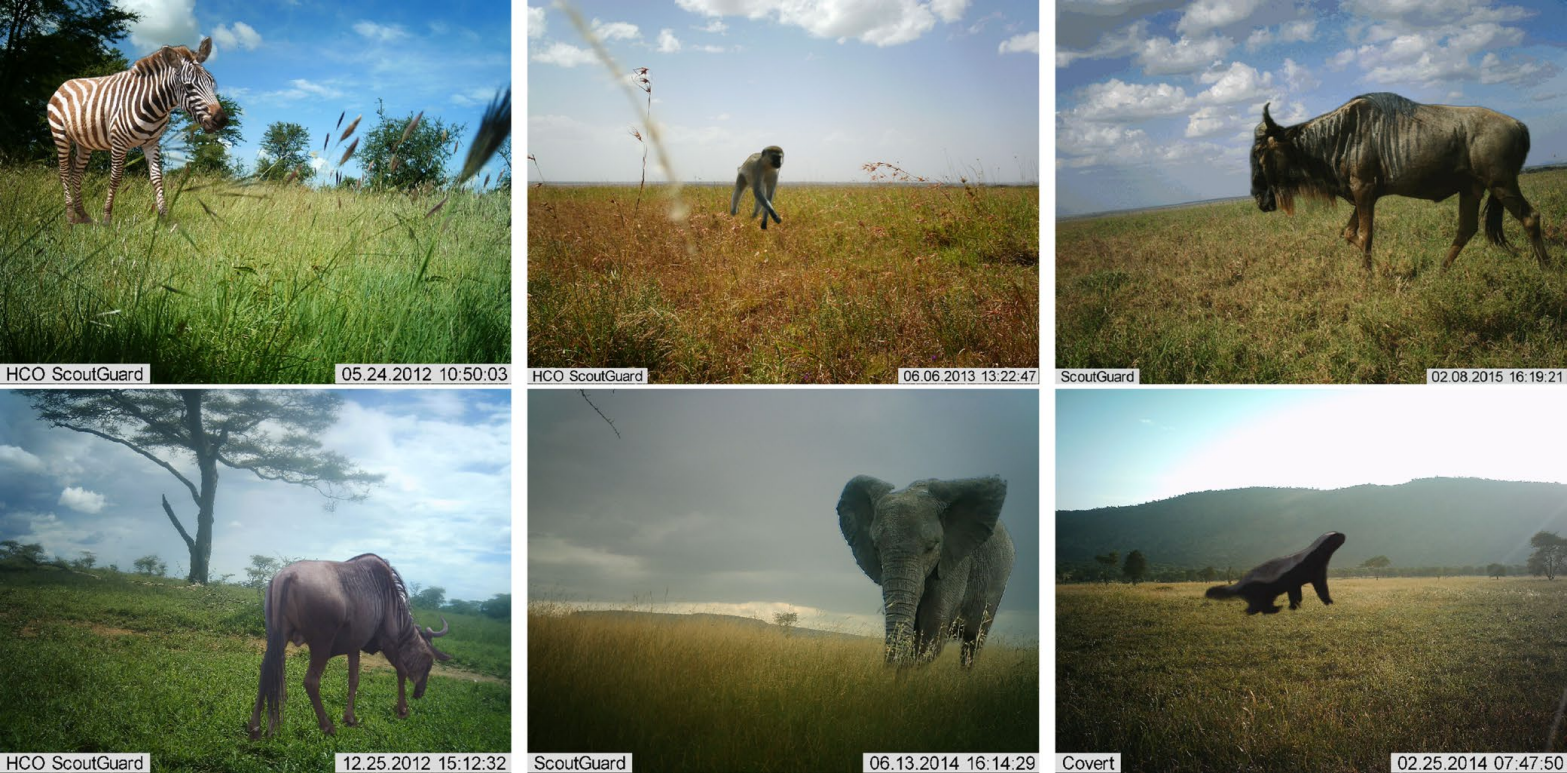
Examples of segments copy‐pasted onto empty backgrounds.

Copy‐paste augmentation involves pasting images of animals that have no background, onto backgrounds that contain no animals, to create new images (Figure 1). Images of animals that have had their backgrounds removed are called segments. To automate segmentation, we use *U* ^2^‐NET, a convolutional neural network for image segmentation and background removal (Qin et al. 2020). We use this ‘out of the box’, and not specifically trained on our dataset. To prevent having multiple animals in the same segment, we focus on images with a single bounding box. We use the bounding boxes included as part of the dataset (see details in ‘Data’). For each bounding box, we extend the bounding box by 10%, crop the image to the bounding box and use the pretrained *U* ^2^‐NET to remove the background. After removing the background, we tighten the bounding box to match the silhouette of the segment. Following segmentation, we exclude segments that contain less than 30% nontransparent pixels. This is because these segments are usually cases where the segmentation algorithm has made errors, such as selecting the background instead of the animal, or where animals are missing most of their bodies. We also discard segments that are smaller than 50 × 50 pixels, as these are often inappropriate for use as targets for pasting, such as animals very far away, animals occluded by too much foreground, or animals where most of the body was removed as it was not detected as an object by *U* ^2^‐NET. The reason for such a drastic filtering is to reduce the amount of manual filtering to be done. To ensure quality, we manually filter the resulting 5235 segments to remove any remaining erroneous segments, leaving 3585 usable segments. Figure 2 shows six example segments automatically extracted and copy‐pasted onto empty backgrounds.

### 2.4 Automated Copy Pasting of Animals

To generate augmented datasets, we use the segmented animals from images randomly sampled from the raw dataset and from the training locations of that iteration. For each class (species), we create as many copy‐paste images as required, depending on the number of usable segments available for that class. For each image, the segment is randomly shrunk or expanded by ±5%, rotated by ±5°, and flipped horizontally (mirrored in the y‐axis). We choose a random *x* and *y* coordinate as the location to paste the segment, such that the bottom of the segment is in the bottom half of the background being pasted onto. Segments are pasted onto empty background images chosen randomly from the test locations of that iteration.

### 2.5 Evaluation

In computer vision, AI models typically fall into two categories, classifiers and object detectors. Classifiers assign labels to images, while detectors enable the localisation and identification of individual objects within an image. Camera trap images often show multiple animals within a single image. Therefore, for this study, we evaluated the use of detectors. This is in line with other studies in the field (e.g., Norouzzadeh et al. (2021), Beery et al. (2019)).

The software package we use for object detection, YOLOv5 (Jocher 2020), produces mean average precision metrics (*mAP*). The mean average precision is the average precision over all classes detected and the average precision is calculated based on precision and recall.

We used the YOLOv5s model to enable our analysis (Monte Carlo evaluation; experiments systematically varying training data) in the presence of computational constraints. While this is a relatively lightweight model, the purpose of our paper is not to benchmark model performance per se, but to investigate the impact of copy‐paste augmentation strategies on object detection performance. We believe the benefits of augmentation are likely to generalise across architectures, though this is a useful area for future research.

We evaluate the performance of our algorithms using the mean delta mean average precision (mAP):

(1) $$\bar{\Delta \mathrm{mAP}}=\frac{1}{k}\sum_{i=1}^{i=k}\mathrm{mAP}\left(i,\mathrm{raw}+\mathrm{aug}\right)-\mathrm{mAP}\left(i,\mathrm{raw}\right)$$

We define the $\bar{\Delta \mathrm{mAP}}$ as the mean over *k* Monte Carlo iterations of the difference between the *mAP* resulting from training on an augmented dataset and the *mAP* resulting from the training on the raw dataset in the same iteration. *mAP* is a widely used metric for evaluating object detection algorithms, derived from the confusion matrix.

We carried out two sets of experiments: (i) a traditional experiment using 500 images per class in the raw training set; and (ii) a few‐shot learning approach using very small numbers of images per class in the raw training set (between 1 and 8 images; see Table S2 for details). The two sets of experiments followed the same method as described above, and differed only in the number of images used for training.

Figure S2 gives the versions of all software packages used in this analysis to facilitate reproducibility.

## 3 Results

### 3.1 500 Images Per Class

**FIGURE 3:**
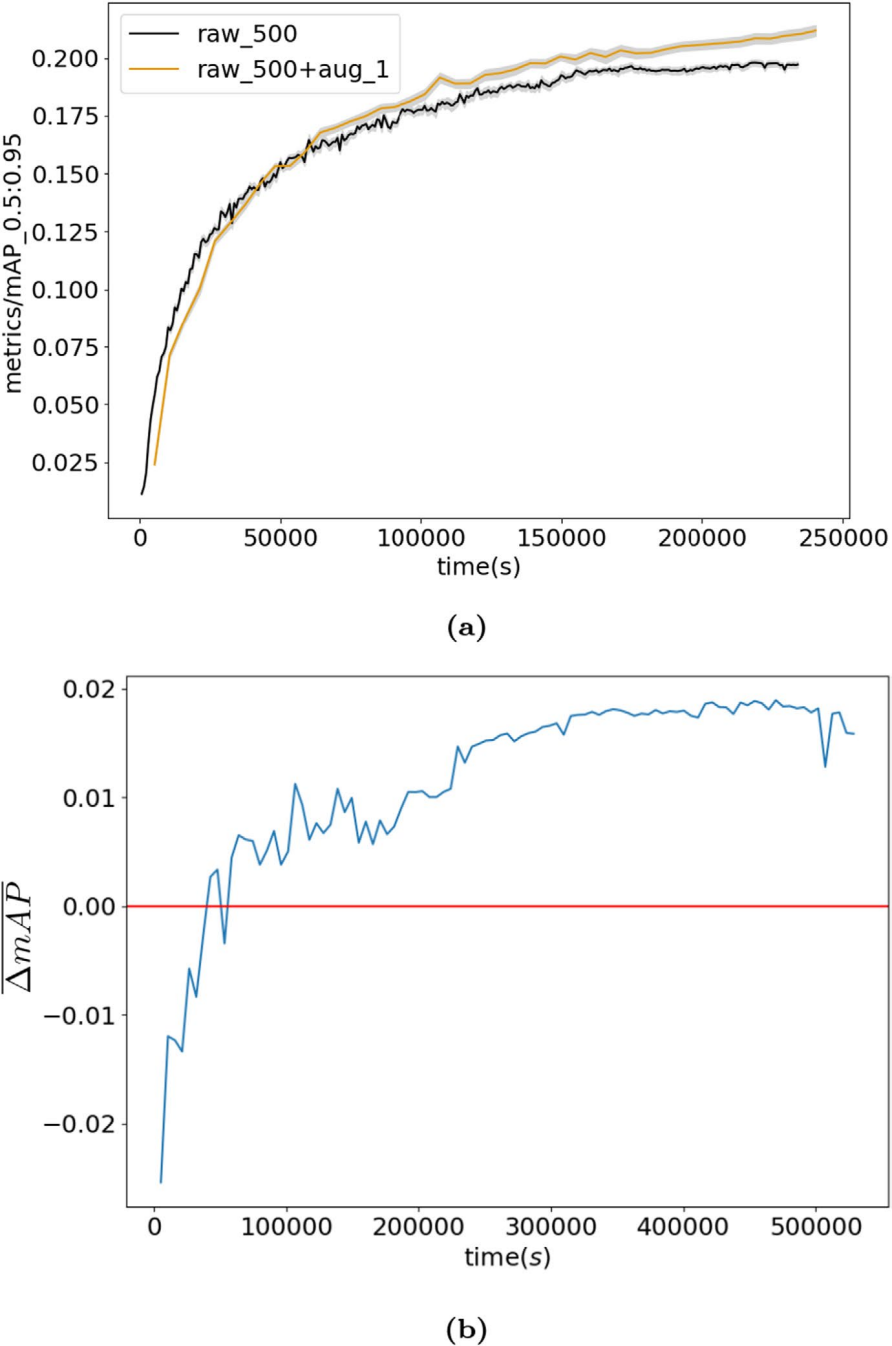
(a) The mean mAP for raw and augmented datasets throughout the training, calculated on the validation set, averaged over 10 Monte Carlo samples. The raw dataset contained 500 images per class (‘raw_500’) and the augmented dataset contained 500 raw and 500 generated images per class (an augmentation factor of 1, ‘raw_500 + aug_1’). The mAP [0.5:0.95] *y*‐axis label represents the number of true positives over the total number of true positives and false positives with an intersection over union (IoU) between 0.5 and 0.95. The IoU relates to the overlap of the original bounding boxes and the detected ones. Grey bands represent the standard error of the mean. (b) $\bar{\Delta \mathrm{mAP}}$ training results on 500 raw images with an augmentation factor of 1 over the *k* = 10 Monte Carlo samples.

When training with 500 raw images and 500 augmented images per class, $\bar{\Delta \mathrm{mAP}}$ is positive. This means that the mean average precision is higher when raw and augmented images are used (when copy‐paste augmentation was used), compared to when just raw images are used (without copy‐paste augmentation). Figure 3 shows the mean mAP for raw and augmented datasets throughout the training, calculated on the validation set. The mean $\bar{\Delta \mathrm{mAP}}$ over 10 iterations is 0.0156 ± 0.00496 (SE) when evaluated on the test sets (ranging between 10 and 15,000 images), corresponding to an 8% ± 2% gain in accuracy.

**FIGURE 4:**
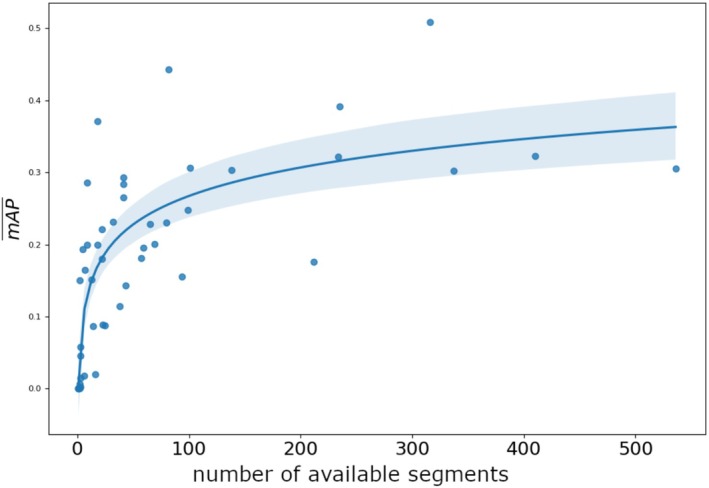
Correlation between the $\bar{\mathrm{mAP}}$ and the number of usable segments per class over 10 iterations. Each point represents a class (species).

Figure 4 shows the relationship between the number of usable segments per species and the mAP test results. We find a significant correlation of the form $y∼\log\left(x\right)$ (estimate = 0.0569, *p* < 0.001, *R* ^2^ = 0.597).

**FIGURE 5:**
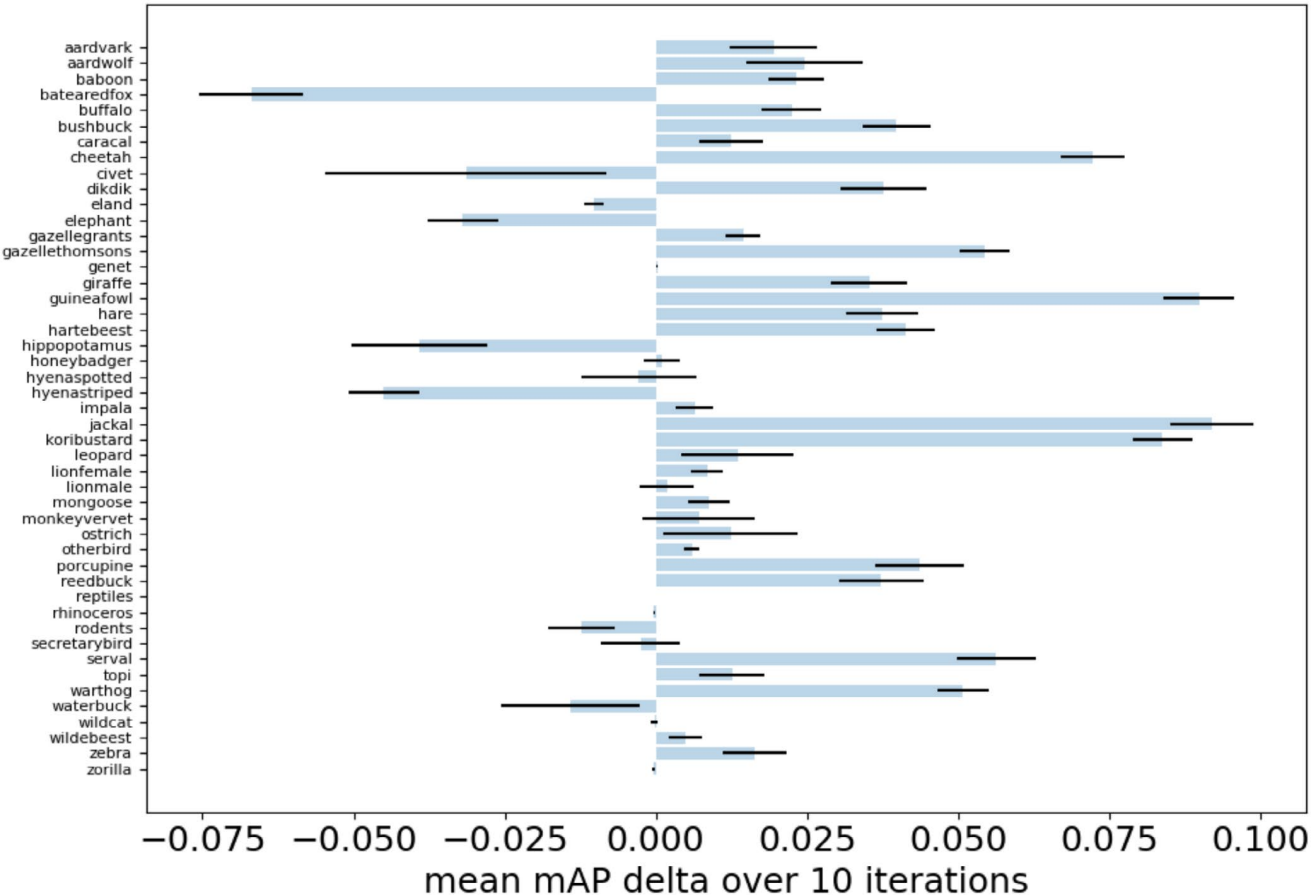
Horizontal bar plot of the $\bar{\Delta \mathrm{mAP}}$ per class over 10 iterations when evaluated on the test sets with the best weights at 300 epochs for the raw dataset, and 50 epochs for the raw + aug dataset (an equivalent total training time).

Figure 5 shows model performance for each class. For the vast majority of classes, model performance was higher with copy‐paste augmentation. The jackal, guinea fowl, and kori bustard gained the most from copy‐paste augmentation. However, for eight (17%) classes (bat‐eared fox, civet, eland, elephant, hippopotamus, striped hyenas, rodents, and waterbuck), model performance was substantially lower with copy‐paste augmentation compared to when only raw images were used.

### 3.2 Few‐Shot Learning

**FIGURE 6:**
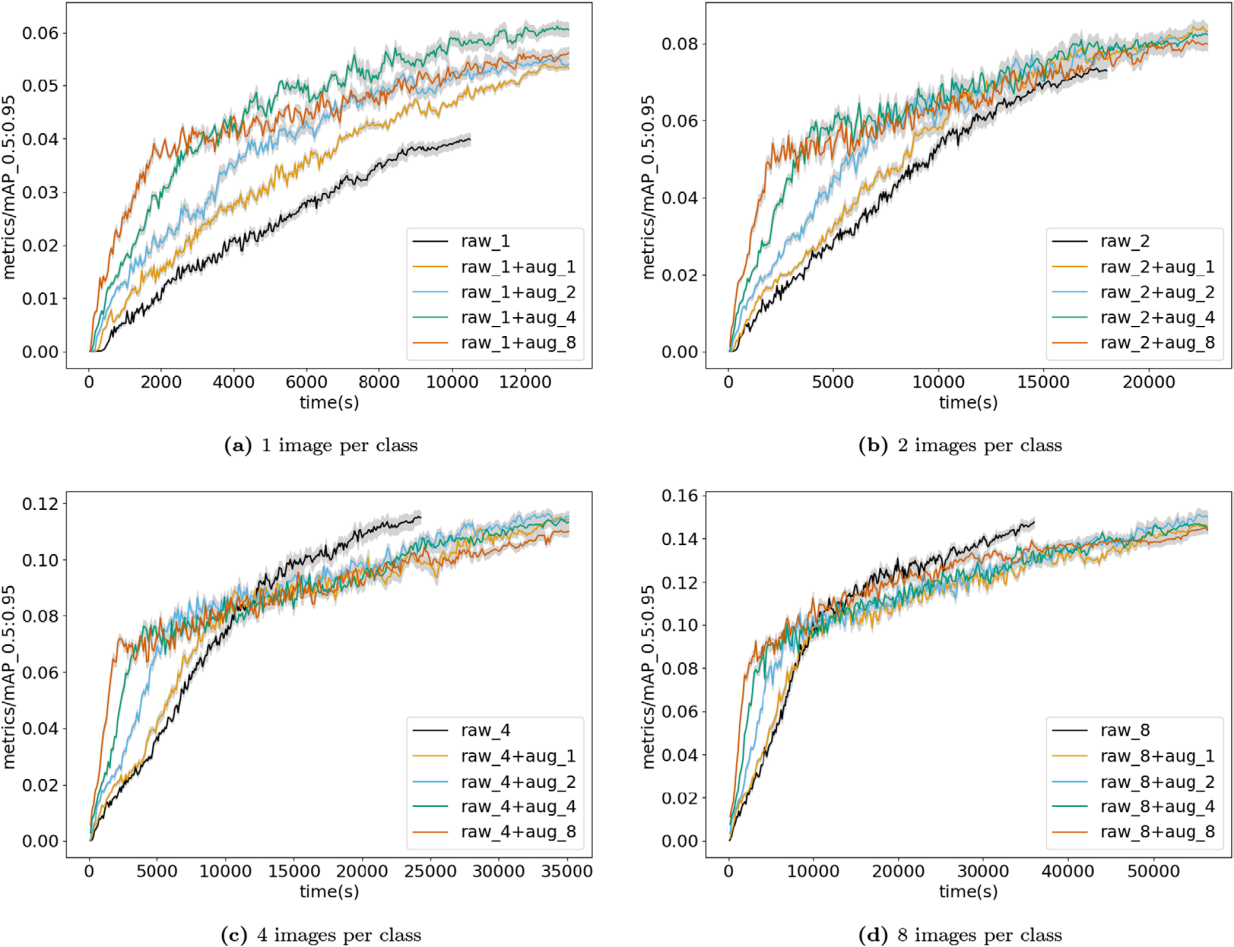
Results of few‐shot learning validated on a 10 images per class validation set at each epoch, up to 300 epochs. Copy‐paste augmentation improves accuracy when 1 or 2 images per class are used, but worsens accuracy when 4 or 8 images per class are used. Results are projected onto training time. Results are shown for 1, 2, 4, 8 images per class and augmentation factors of 1, 2, 4, 8. ‘raw_*n*’ by itself shows the performance of the model when trained on just raw images. ‘raw_*n* + aug_*n*’ shows the performance of models trained on augmented datasets containing raw and generated images. ‘raw_*n*’ indicates *n* raw images per class in a dataset. ‘aug_*m*’ indicates an augmentation factor of *m*. An augmentation factor of 1 means the augmented dataset contains an equal number of raw and generated images; an augmentation factor of 2, means the augmented set contains twice as many generated images as raw images, and so on. For example, ‘raw_8 + aug_8’ shows the performance of a model trained on 72 images, comprising 8 raw images and 64 generated images. See Table S2 for full details of dataset sizes. The mAP [0.5:0.95] *y*‐axis label represents the number of true positives over the total number of true positives and false positives with an intersection over union (IoU) between 0.5 and 0.95. The IoU relates to the overlap of the original bounding boxes and the detected ones.

**FIGURE 7:**
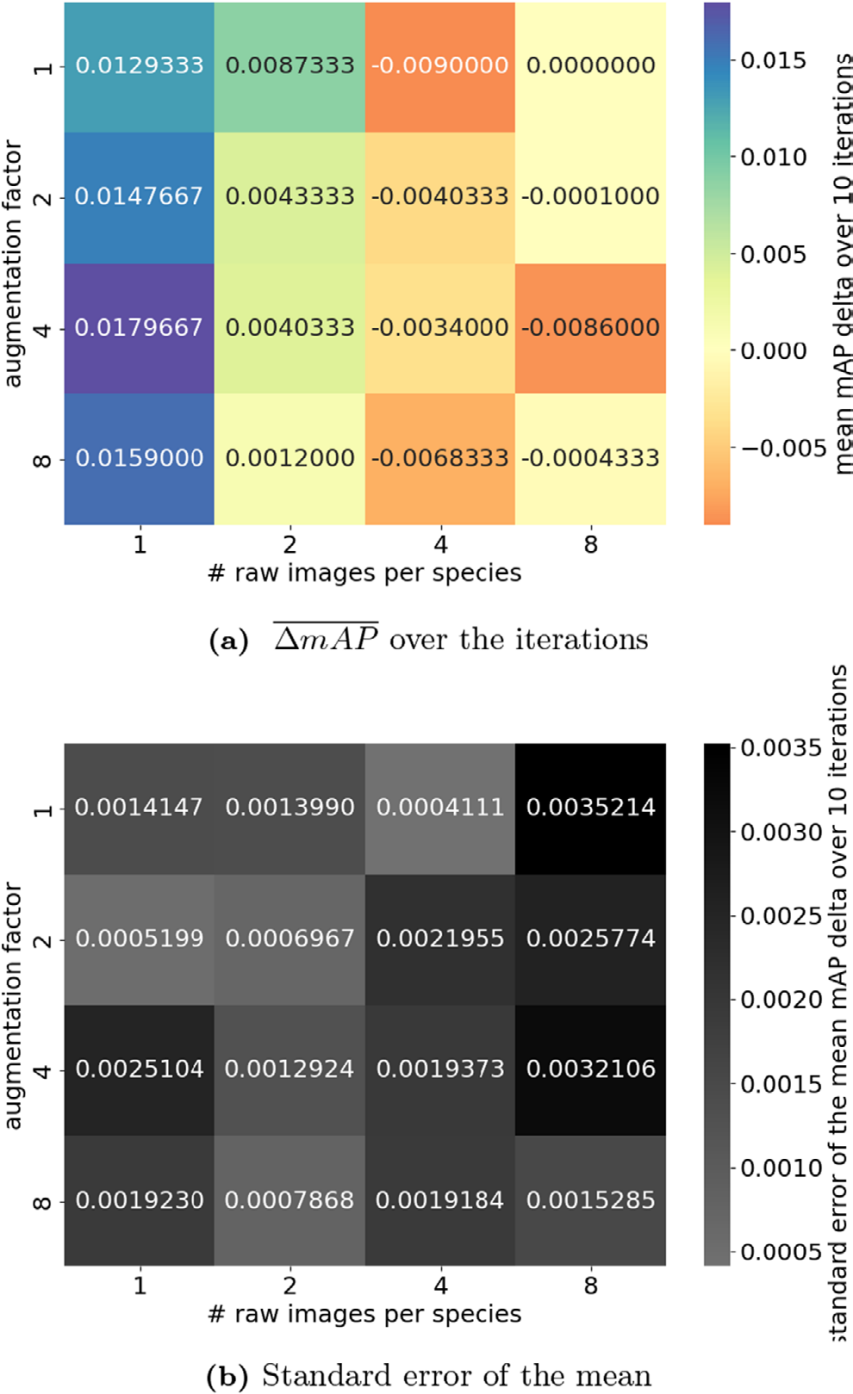
Aggregated results of the few‐shot learning experiment, showing (a) mean change in mAP and (b) standard error of those means, for different numbers of raw images and augmentation factors.

Model performance when using very small amounts of training data was mixed (Figure 6). When using 1 or 2 images per class, copy‐paste augmentation improved model performance. When 4 or 8 images per class were used, copy‐paste augmentation appeared to worsen model performance (Figure 6). Figure 7 shows $\bar{\Delta \mathrm{mAP}}$ and standard errors from the few‐shot learning approach, evaluated on each iteration's test sets. Results per class are given in Figure S1.

## 4 Discussion

We find that copy‐paste augmentation improves the ability of AI models to identify species in camera trap images from unseen locations. Specifically, we found that using copy‐paste augmentation to double the number of training images per species improved performance by 8% ± 2%.

There are two main augmentation strategies in computer vision: (1) transforming existing images through processes like flipping, rotating, and cropping (Schneider et al. 2020; Shorten and Khoshgoftaar 2019), and (2) generating new, artificial images (Barile et al. 2021; Garcea et al. 2023; Shorten and Khoshgoftaar 2019). While fields like medicine frequently adopt the latter (Garcea et al. 2023), biodiversity science has lagged behind and primarily uses basic image transformations (Schneider et al. 2020) or no augmentation at all (Norouzzadeh et al. 2021). While the reasons for this are unclear, it could be because camera trap images have a high background complexity and large variations in foreground animals, making it challenging to accurately segment animals from the background.

Our results suggest a promising future for augmentation with artificial images in biodiversity monitoring. Specifically, copy‐paste augmentation can help address two main challenges (Schneider et al. 2020). First, it helps improve transferability to new locations. This ‘domain shift’ is a major challenge: in a recent study, the best performing model achieved 95.6% accuracy when tested on locations seen during training, but only 68.7% accuracy when tested on unseen locations. In general, neural networks perform best when the testing and training data are similar (Goodfellow 2016; LeCun et al. 2015). However, this is rarely the case in conservation, where users will often want to identify species in images in new locations that have different backgrounds from those seen in training (Meek et al. 2013). Here we show that augmentation improves model performance on unseen locations for ‘free’; that is, higher transferability can be achieved via augmentation without the need for any additional data.

Second, augmentation helps address the issue of unbalanced datasets. Camera trap datasets have highly skewed frequency distributions across species, with a few species having large numbers of images and many species having few images. Accurately classifying species with few images to train on poses a significant challenge for species identification AIs, as models typically require large amounts of data for training (Norouzzadeh et al. 2018; Tabak et al. 2019; Willi et al. 2019). Here we show that copy‐paste augmentation improves performance in classes with only 500 images, suggesting that it is a valid strategy for rebalancing datasets and addressing this problem.

However, augmentation approaches are not immune from data demands. As Figure 3 shows, model performance was higher for species with a larger number of available segments. This could result in a ‘rich get richer’ effect, where performance is higher for frequently occurring species that have many available segments to paste, compared to data‐sparse species for which a less diverse set of augmented images can be created. Thus, while copy‐paste augmentation can improve performance for species that have a low numbers of available images from which to source segments, it may still not solve the issue of unbalanced data for species with extremely low numbers images (e.g., one); a minimum number of available segments is needed to produce an augmented dataset with sufficient diversity to improve performance. Encouragingly, however, the non‐linear relationship between number of segments and $\bar{\mathrm{mAP}}$ (Figure 3) suggests that, initially, large increases in performance are achieved for small increases in number of segments, with relatively few segments needed to approach peak performance (∼50 segments). Further research across datasets is needed to confirm if this pattern is general.

While, for the vast majority of species, copy‐paste augmentation improved performance, for eight species (17%) it substantially decreased performance. One possible reason for this is that all but one (elephant) of these negative‐performing species are to some degree nocturnal or crepuscular: the bat‐eared fox, civet, striped hyena, and many rodents are nocturnal, the common eland is crepuscular, and hippopotamuses and waterbucks are often active at night. This means it is likely that the segments of these animals are from images captured at night. We did not synchronise times between segments and backgrounds; thus, night segments of these species could have been pasted onto day backgrounds, resulting in unusual images that the model performed worse on. This could have been compounded by AI models' generally lower performance on night camera trap images due to greyscale, grain, glare from flash and shorter viewing distance (Mitterwallner et al. 2024). However, given that our augmentation approach improved performance substantially for other nocturnal species, such as aardvark and porcupine, this cannot fully explain our results. Another possible explanation is that these species have some morphological or behavioural traits that are not captured through simple duplication; these may be species that require a greater diversity of images for improved model performance. Alternatively, these species' traits might mean the segmentation algorithm performed worse, perhaps removing too much or too little of the source image, resulting in subtly erroneous segments that lose necessary detail. Finally, in our analysis, animals are pasted randomly onto random backgrounds (with the constraint that the bottom of the segment is in the bottom half of the image). In reality, animals have relatively fixed territorial ranges or activity spaces, and therefore random pasting could result in unrealistic image compositions for some species which, in turn, could degrade model performance. Future work could investigate ‘smart pasting,’ where animal segments are placed non‐randomly in an image, such as birds being placed in the sky or elephants being scaled correctly relative to the background. This is a non‐trivial extension of our work but is an interesting direction for future research.

We found that augmentation had a mixed impact in a few‐shot learning context. Augmentation substantially improved model performance when there were only one or two raw images per class. However, when four or eight images per class were used, augmentation reduced performance. The augmentation factor (number of augmented images) seemed to have little impact on performance, with the exception of when only one raw image per class was used: in this analysis, augmentation factor four performed best, followed by eight, two, then one. Taken together, these results are hard to fully explain. Data augmentation is an established few‐shot method (Liu et al. 2022; Tian et al. 2024) and has previously shown consistent improvements in model performance across a range of dataset sizes (Ghiasi et al. 2021b), and thus we would expect consistent improvement. Notably, in all cases, augmented models perform better than raw‐only models in the early parts of training (Figure 5). Simply increasing computational resources could therefore produce improved results; for example, training the models for a longer time period or running more iterations of each analysis to reduce stochastic effects. Alternatively, drastically increasing the number of augmented images could produce improvements—our analysis includes a maximum of 64 augmented images (eight raw images with an augmentation factor of 8). Testing, for example, eight raw images with thousands of augmented images could be an interesting future direction to establish the limits of this approach. Further research is needed into the value of copy‐paste augmentation in a few‐shot context before its utility can be fully assessed. However, the results shown here for one or two images per class show there is significant promise of the approach.

Our research demonstrates the potential for artificial image augmentation in biodiversity monitoring, and thus opens promising avenues for future research. First, it is important to validate our approach in other datasets that span a wide range of species, habitat types, and locations.

Second, due to the imperfect segmentation algorithm, we had to manually filter segments to ensure quality. This is time‐consuming and therefore we tried to programmatically ensure segments would be high quality, to reduce the amount of manual filtering required (see Methods). This led us to exclude a large number of images. In the future, better segmentation methods trained specifically for segmenting animals could negate the need for manual filtering and therefore allow more images to be retained. Moreover, without manual filtering, our approach could be scaled to very large numbers of synthetic images. Having demonstrated that copy‐paste augmentation works as a proof of concept, fully automating the segmentation step is an important direction for future research.

Third, there are several ways the copy‐paste approach could be improved to potentially achieve higher performance. For example, time could be synchronised between segments and backgrounds so that segments are pasted onto backgrounds of an approximately similar time of day, creating a better match between segment and background lighting conditions. Methods for ‘smart’ pasting could also be developed to ensure that segments are pasted onto backgrounds in a sensible way that results in realistic images; for example, ensuring land animals are not pasted onto the sky. A more complex solution could ensure animals are pasted at locations on the background such that the resulting images look natural. Currently, our approach can result in nonsensical images: for example, pasting elephant segments onto backgrounds with blades of grass in the foreground, resulting in images where elephants appear smaller than blades of grass. Improving pasting methods and assessing whether this increases performance is an important direction for future research.

Fourth, although the model trained on raw and augmented images performed better than the model trained on raw images alone, it is important to note that the ‘raw_500’ model contained twice as many images as the ‘raw_500 + aug_1’ model. Our intention with this experiment was to evaluate the practical impact of incorporating synthetic data into a limited real‐world dataset. In applied settings, practitioners are not constrained to maintaining a fixed dataset size, but instead would aim to improve performance by leveraging any available data, including synthetic samples. Therefore, our goal was to test whether augmenting a small real dataset with synthetic images would lead to meaningful performance improvements. We note that this approach is used in other studies of synthetic image augmentation in biodiversity monitoring (e.g., Beery et al. (2020)). However, it would be an interesting avenue for future research to isolate whether the benefit of augmentation comes from the increased number of images or the method of augmentation per se.

Fifth, it would be interesting to investigate whether animal segments from sources external to the dataset (e.g., images from an internet search) could be used as part of the copy and paste method in addition to, or instead of, animal segments from the camera trap images. This could provide a vast source of animal segment data to increase the diversity of the augmented dataset even further.

Overall, we show that copy‐paste augmentation shows significant promise as a way to address key challenges in biodiversity monitoring AI. Specifically, it improves transferability to unseen locations and can help balance typical long‐tailed ecological camera trap data. Ecologists and conservationists must move beyond just simple image transformations and embrace artificial images as another tool for augmentation.

## Author Contributions

**Cédric S. Mesnage:** conceptualization (equal), data curation (lead), formal analysis (lead), software (lead), validation (lead), visualization (lead), writing – original draft (lead), writing – review and editing (equal). **Andrew Corbett:** conceptualization (equal). **Jake Curry:** conceptualization (equal). **Sareh Rowlands:** conceptualization (equal). **Anjan Dutta:** conceptualization (equal), funding acquisition (equal), writing – review and editing (equal). **Richard Everson:** conceptualization (equal), writing – review and editing (equal). **Benno I. Simmons:** conceptualization (equal), funding acquisition (equal), supervision (lead), writing – original draft (equal), writing – review and editing (equal).

## Conflicts of Interest

The authors declare no conflicts of interest.

## Supporting information


**Figure S1:** Heatmap of the ∆mAP per species at 300 epochs.
**Figure S2:** Software packages versions.
**Table S1:** List of removed locations per season due to are sizing issue in the original dataset.
**Table S2:** Number of images used in each few‐shot learning experiment.

## Data Availability

The Serengeti dataset is publicly accessible at https://lila.science/datasets/snapshot‐serengeti. Our code is uploaded as a zip file for review at https://doi.org/10.5281/zenodo.13821558.
