# Supplementary material for: Copy‐Paste Augmentation Improves Automatic Species Identification in Camera Trap Images
Source: Ecol Evol. 2025 Nov 5;15(11):e72357. doi: 10.1002/ece3.72357 (PMC12588685; doi:10.1002/ece3.72357)
Supplement: Supplementary file 1 — Figure S1: Heatmap of the ∆mAP per species at 300 epochs. Figure S2: Software packages versions. Table S1: List of removed locations per season due to are sizing issue in the original dataset. Table S2: Number of images used in each few‐shot learning experiment. [file ECE3-15-e72357-s001.zip › supinfo.pdf]

Appendix for

Copy-paste augmentation improves the accuracy of automated species identification in camera trap images

1. Institute for Data Science and Artificial Intelligence, University of Exeter, Exeter, UK
2. Centre for Ecology and Conservation, University of Exeter, Penryn, UK
3. Department of Computer Science, University of Exeter, Exeter, UK
4. Institute for People-Centred Artificial Intelligence, University of Surrey, Guildford, UK

* Corresponding author: bsimmons.research@gmail.com

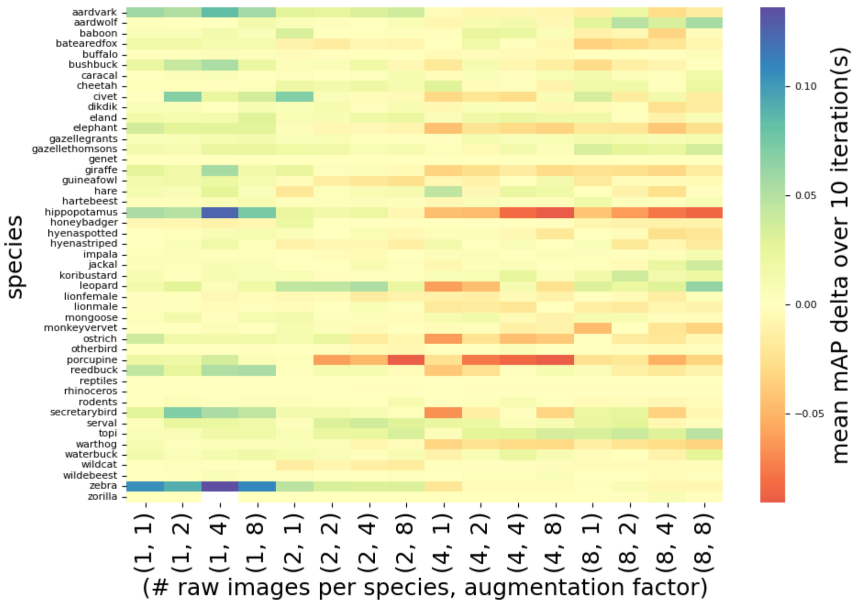

Figure S1: Heatmap of the $\overline{\Delta mAP}$ per species at 300 epochs

Season	Location	Season	Location
S2	E01	S6	J09
S3	E11	S6	D08
S3	J08	S6	U13
S4	R11	S6	S12
S4	E11	S6	P10
S4	E01	S6	L13
S5	G07	S6	S11
S5	L04	S6	F10
S5	E09	S6	J13
S5	E12	S6	E12
S5	C11	S6	E02
S5	B09	S6	C07
S5	D10	S6	C11
S5	I05	S6	K09
S5	J08	S6	E03
S6	G07	S6	R13
S6	G10	S6	B09
S6	E10	S6	Q10
S6	H10	S6	O11
S6	D05	S6	D10
S6	O13	S6	O12
S6	C10	S6	I05
S6	L04	S6	J08
S6	E09	S6	R12

Table S1: List of removed locations per season due to a resizing issue in the original dataset.

Name	Number of raw images	Number of generated images
raw_1	1	0
raw_1+aug_1	1	1
raw_1+aug_2	1	2
raw_1+aug_4	1	4
raw_1+aug_8	1	8
raw_2	2	0
raw_2+aug_1	2	2
raw_2+aug_2	2	4
raw_2+aug_4	2	8
raw_2+aug_8	2	16
raw_4	4	0
raw_4+aug_1	4	1
raw_4+aug_2	4	8
raw_4+aug_4	4	16
raw_4+aug_8	4	32
raw_8	8	0
raw_8+aug_1	8	8
raw_8+aug_2	8	16
raw_8+aug_4	8	32
raw_8+aug_8	8	64

Table S2: Number of images used in each few-shot learning experiment.

```
yolov5==6.2.0
# Base ----------------------------------------
matplotlib>=3.2.2
numpy>=1.18.5
opencv-python>=4.1.1
Pillow>=7.1.2
PyYAML>=5.3.1
requests>=2.23.0
scipy>=1.4.1
torch>=1.7.0  # see https://pytorch.org/get-started/locally/ (recommended)
torchvision>=0.8.1
tqdm>=4.64.0
# protobuf<=3.20.1  # https://github.com/ultralytics/yolov5/issues/8012

# Logging -------------------------------------
tensorboard>=2.4.1
# clearml>=1.2.0
# comet

# Plotting ------------------------------------
pandas>=1.1.4
seaborn>=0.11.0
```

Figure S2: Software packages versions
