# Supplementary figures and images for: Copy‐Paste Augmentation Improves Automatic Species Identification in Camera Trap Images
Source: Ecol Evol. 2025 Nov 5;15(11):e72357. doi: 10.1002/ece3.72357 (PMC12588685; doi:10.1002/ece3.72357)

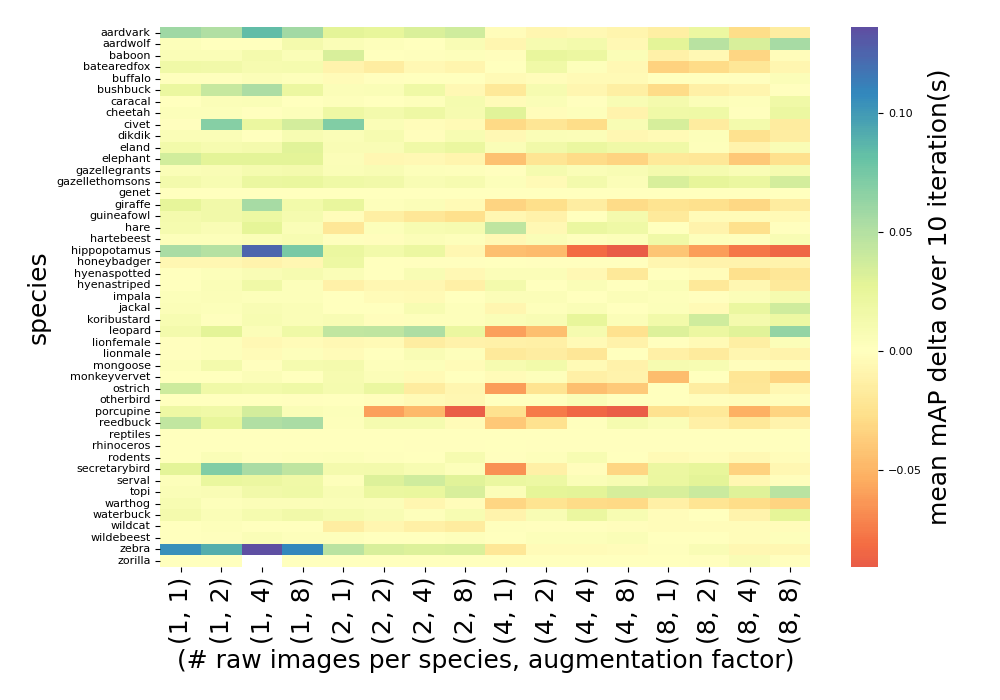

Supplement: Supplementary file 1 — Figure S1: Heatmap of the ∆mAP per species at 300 epochs. Figure S2: Software packages versions. Table S1: List of removed locations per season due to are sizing issue in the original dataset. Table S2: Number of images used in each few‐shot learning experiment. [file ECE3-15-e72357-s001.zip › species_1_2_4_8_1_2_4_8.png]
